# The relationship between minimum inhibitory concentration and 28 day mortality in patients with a Gram-negative bloodstream infection: an analysis of data from a cohort study (BSI-FOO)

**DOI:** 10.1093/jacamr/dlad009

**Published:** 2023-02-01

**Authors:** Rebecca N Evans, Jessica Harris, Chris A Rogers, Alasdair P MacGowan

**Affiliations:** Bristol Trials Centre, Bristol Medical School, University of Bristol, 1-5 Whiteladies Road, Clifton, BS8 1NU Bristol, UK; Bristol Trials Centre, Bristol Medical School, University of Bristol, 1-5 Whiteladies Road, Clifton, BS8 1NU Bristol, UK; Bristol Trials Centre, Bristol Medical School, University of Bristol, 1-5 Whiteladies Road, Clifton, BS8 1NU Bristol, UK; Bristol Centre for Antimicrobial Research & Evaluation (BCARE), Infection Sciences, Pathology, North Bristol NHS Trust, Bristol, UK

## Abstract

**Objectives:**

To explore the association between MIC/EUCAST breakpoint ratio and 28 day mortality in patients with a Gram-negative bloodstream infection (BSI).

**Methods:**

Using data from the Bloodstream Infection—Focus on Outcomes (BSI-FOO) observational study, we defined an average MIC/EUCAST breakpoint ratio that was updated daily to reflect changes in treatment in the first 7 days after blood culture. Cox regression analysis was performed to estimate the association between MIC/EUCAST breakpoint ratio and mortality, adjusting for organism and a risk score calculated using potential confounding variables. The primary outcome was 28 day all-cause mortality from the date of blood culture.

**Results:**

Of the 1903 study participants, 514 met the eligibility criteria and were included in the analysis (*n* = 357 *Escherichia coli*, *n* = 6 *Klebsiella* and *n* = 151 *Pseudomonas aeruginosa*). The average age was 74.0 years (IQR 60.0–82.0). The mortality rate varied from 11.1% (in patients treated with an average MIC/EUCAST breakpoint ratio of 1) to 27.6% (in patients treated with antibiotics with an average MIC/EUCAST breakpoint ratio >1). After adjusting for risk score and organism, MIC/EUCAST breakpoint ratio was not associated with 28 day mortality (*P* = 0.148).

**Conclusions:**

In an adjusted model controlling for potential confounding variables, there was no evidence to suggest a relationship between MIC/EUCAST breakpoint ratio and 28 day mortality in patients with a Gram-negative BSI.

## Introduction

Susceptibility results from MIC tests play a key role in treatment decision-making, and guide the clinician as to which treatments patients are likely to respond to. However, there is a grey area around the breakpoint where strains may not respond as well, even though the MIC is within the susceptible range. It has been shown in some studies that high MICs in the susceptibility range have worse outcomes, and this may help clinicians explain a slower response to treatment for some patients or suggest benefits of including an index of the degree of susceptibility to the treatments.^1–3^

However, studies to date have focused on the MIC of a particular drug and there is a lack of research exploring the overall relationship between MIC and patient outcomes.

In this report we explore whether MIC values closer to the EUCAST breakpoints are associated with worse outcomes than lower MIC values in infections caused by Gram-negative bloodstream infection (*Escherichia coli*, *Klebsiella* or *Pseudomonas aeruginosa*).

## Materials and methods

### Data source

This study is a *post hoc* analysis of data from the Bloodstream Infections—Focus on Outcomes (BSI-FOO) observational study (described in detail elsewhere).^4^ Briefly, BSI-FOO was a multicentre cohort study of 1903 hospitalized adults with a BSI conducted between 2010 and 2012 with the primary aim of identifying modifiable risk factors for 28 day mortality. The population included adults receiving inpatient NHS hospital care at one of five participating centres in England and Wales and having a clinically significant BSI with an organism in one or more of the following six target groups: MRSA, MSSA, non-ESBL-producing *E. coli*, any ESBL-producing member of the family Enterobacteriaceae, *P. aeruginosa* and any species of *Candida*. Organism samples were sent by the five contributing centres to a central laboratory, where they were tested against a selected range of antimicrobials.

### Study population

All BSI-FOO participants with a Gram-negative BSI were considered for inclusion in this study; this included *E. coli, Klebsiella* and *P. aeruginosa*. Some isolates were not sent for central testing, generally because the laboratories had failed to retain them. Therefore, MIC information was not available for all isolates. Patients who were in receipt of a drug where MIC data were not available or if they were in receipt of any drugs where MIC was not tested were excluded. Polymicrobial infections and repeat episodes were also excluded.

### MIC

MICs were measured using the CLSI M7-A8 agar dilution method with Mueller–Hinton agar^5^. *E. coli* and *Klebsiella* isolates were tested against co-amoxiclav, ampicillin, ceftriaxone, ciprofloxacin, ertapenem, gentamicin, meropenem and piperacillin/tazobactam. P. aeruginosa isolates were tested against ceftazidime, ciprofloxacin, colistin, gentamicin, meropenem and piperacillin tazobactam. EUCAST v9.0 breakpoints were used for susceptibility classification.^6^

The MICs cannot be directly compared between drugs because the breakpoint for each drug is different. Therefore a ratio of MIC to EUCAST breakpoint (MIC/EUCAST breakpoint) was defined. When susceptible to an antibiotic, the strain has a ratio of ≤1, and when resistant, the strain has a ratio of >1. Only the MIC of drugs administered in the first 7 days post blood culture were explored as this period can be considered the most critical for choice and timing of therapy.

### Outcome measures

The primary outcome was 28 day all-cause mortality from date of blood culture.

### Statistical analyses

Demographics, comorbidities and medical history were summarized by MIC/EUCAST breakpoint ratio category on the last day of follow-up. Continuous data were summarized using mean and SD (or median and IQR if distributions were skewed) and categorical data as numbers and percentages.

Cox regression analysis was performed to estimate the association between MIC/EUCAST breakpoint ratio and mortality, where the MIC/EUCAST breakpoint ratio was categorized into the following groups: <0.125 [susceptible (S)], 0.125 to <0.25 (S), 0.25 to <0.5 (S), 0.5 to <1 (S), 1 (S), >1 [resistant (R)]. Drugs administered can vary on a daily basis, therefore to allow for changes in treatment, the infection episodes were split (using the -*stsplit*- Stata command^7^) at daily intervals and the median MIC/EUCAST breakpoint ratio was calculated for each day using the MICs/breakpoint ratios of drugs administered up until that day (see example calculation in Table 1). Estimates were adjusted for organism and a risk score calculated from a Cox regression model of baseline variables associated with mortality such as patient demographics and long-term comorbidities; the variables included in the risk score are given in Table S1, available as Supplementary data at *JAC-AMR* Online, and the derivation of the risk score is described elsewhere.^4^

**Table 1.** Example calculation of the cumulative average MIC/EUCAST breakpoint ratio

	MIC/EUCAST breakpoint ratio				
Day	Gentamicin (in receipt on Day 0 and 1)	Piperacillin/tazobactam (in receipt on Day 2 only)	Ceftriaxone (in receipt on Days 2, 3 and 4)	Cumulative average (median)	Calculation
0	1			1	= median of 1
1	1			1	= median of 1, 1
2		0.0625	0.03	0.53125	= median of 1, 1, 0.0625, 0.03
3			0.03	0.0625	= median of 1, 1, 0.0625, 0.03, 0.03
4			0.03	0.04625	= median of 1, 1, 0.0625, 0.03, 0.03, 0.03
5				0.04625	
6				0.04625	
7 to 28				0.04625	

Example is given for a patient with *E. coli* who was in receipt of gentamicin on Days 0 and 1 (MIC = 2 mg/L, EUCAST breakpoint = 2 mg/L), piperacillin/tazobactam on Day 2 (MIC = 0.5 mg/L, EUCAST breakpoint = 8 mg/L) and ceftriaxone on Days 2, 3 and 4 (MIC = 0.03 mg/L, EUCAST breakpoint = 1 mg/L).

All analyses were performed in Stata version 17.0 (StataCorp, LP, College Station, TX, USA).

## Ethics

The research programme on which this work is based was approved by South West 4 Research Ethics Committee (10/HO102/51). The National Information Governance Board approved the use of routinely collected patient data without specific consent and North Bristol NHS Trust acted as sponsor.

## Results

Of the 1903 participants in BSI-FOO, 514 (27.0%) patients met the eligibility criteria and were included in the analysis (*n* = 357 *E. coli*, *n* = 6 *Klebsiella* and *n* = 151 *P. aeruginosa*). The average age was 74.0 years (IQR 60.0–82.0) and approximately 50% were male. Baseline characteristics were broadly similar across the MIC/EUCAST breakpoint categories (Table S2). The most frequently prescribed treatment was piperacillin/tazobactam, prescribed in 72.8% of patients with *E. coli* or *Klebsiella* and 80.8% of patients with *P. aeruginosa* (Table S3).

The mortality rate varied from 11.1% (in patients treated with an average MIC/EUCAST breakpoint ratio of 1) to 27.6% (in patients treated with antibiotics with an average MIC/EUCAST breakpoint ratio of >1). After adjusting for risk score and organism, MIC/EUCAST breakpoint ratio was not associated with 28 day mortality (*P* = 0.148), Table 2.

**Table 2.** Twenty-eight day mortality by cumulative average MIC/EUCAST breakpoint ratio

MIC/EUCAST breakpoint ratio group	*N* ^a^	Died, *n* (%)	HR^b^ (95% CI)
<0.125 (S)	121	26/121 (21.5)	Ref
0.125 to <0.25 (S)	98	14/98 (14.3)	0.56 (0.29–1.11)
0.25 to <0.5 (S)	140	29/140 (20.7)	0.78 (0.43–1.38)
0.5 to <1 (S)	90	19/90 (21.1)	0.61 (0.32–1.14)
1 (S)	36	4/36 (11.1)	0.42 (0.14–1.21)
>1 (R)	29	8/29 (27.6)	1.40 (0.62–3.16)
Overall	514	100/514 (19.5)	

a Based on category of cumulative average MIC/EUCAST breakpoint ratio on last day of follow-up.

b Average MIC/EUCAST breakpoint ratio that was updated daily to reflect changes in treatment in the first 7 days. Estimate adjusted for risk score and organism.

## Discussion

In an adjusted model controlling for potential confounding variables, there was no evidence to suggest a significant relationship between MIC/EUCAST breakpoint ratio and 28 day mortality in patients with a Gram-negative BSI.

Although there have been a number of studies exploring the association between MIC and outcome, to date these have most frequently focused on the MIC of a particular drug in question. In this analysis we attempted to investigate whether there was a general effect of MIC by exploring the MIC/EUCAST breakpoint ratio. There are several reasons that may explain the lack of relationship: (i) the study was underpowered; (ii) if an organism is susceptible to a drug, the drug works in terms of reducing mortality, regardless of the level of susceptibility; (iii) the population is too heterogeneous with multiple drugs administered during follow-up, distorting any relationship.

A study investigating the impact of piperacillin/tazobactam MIC on 30 day mortality in patients with bacteraemia caused by Enterobacteriaceae found no associations.^8^ Similarly, in a propensity score-matched cohort of patients with *P. aeruginosa*, 30 day mortality was not statistically significantly different between patients with low and high cefepime or ceftazidime MIC.^3^ However, a meta-analysis including 115 patients with Enterobacteriaceae BSI treated with a carbapenem found an increase in meropenem MIC was associated with an increase 30 day mortality.^2^ The conflicting results in the literature may be down to the heterogeneity of the populations being studied, such as different pathogens, and also due to the drug in question. Most patients in this analysis were treated with piperacillin/tazobactam, where the MIC has been shown to be unrelated to mortality in other studies.^8,9^ This lack of relationship was also observed in a recently reported study of 1626 patients with *E. coli* bacteraemia, which found there was no evidence that mortality differed for inactive versus active amoxicillin/clavulanate (adjusted HR = 1.27, 95% CI 0.83 to 1.93; *P* = 0.28).^10^

A strength of this analysis is the use of an MIC/EUCAST breakpoint ratio, enabling the MIC of a broad range of drugs to be included. This meant the analysis population did not have to be restricted to patients in receipt of a particular drug, allowing inclusion of a larger sample size and improving generalizability of results. Another strength is that the MIC testing was performed centrally using the same methodology for all isolates, rather than depending individual hospital results, which increases variability.

There are a number of limitations to this analysis. First, as it was observational there is a risk of unmeasured confounding effects. We attempted to adjust for confounding by adjusting for a risk score calculated using baseline variables associated with mortality. Even after adjustment for risk score, there is still chance that residual confounding remains. In addition, potential confounding variables attributable to differential antibiotic exposure can be difficult to measure as they can be based on a clinician’s personal care preferences. Antibiotic dosing and concentration also play an important role in pharmacokinetics/pharmacodynamics and would be important to consider in the design of future studies aiming to explore the relationship between MIC and outcome. As well as residual confounding, there is also the possibility of measurement error within the measurement of MIC. Methods of measuring MIC can sometimes be inaccurate, which could have led to misclassification of the exposure. However, the MIC testing was performed centrally using the same methodology for all isolates in this study, maximizing within-study consistency.

Although we failed to show a relationship with mortality, other studies have found a relationship with treatment failure in other infections.^11^ This outcome was not measured in BSI-FOO but may be a relevant outcome for future studies. In addition, our study was limited to a few organisms (*E. coli*, Klebsiella and *P. aeruginosa*)—a larger study designed to answer this research question across a broader range of enterobacteria and exploring the association between individual antibiotic exposure separately for the different organisms would enable a deeper understanding of the relationship.

It is unlikely that a randomized controlled trial addressing this research question will take place since it is not possible to randomize participants to a specific MIC, therefore observational studies with a protocol designed to answer this question are needed to confirm the findings.

## Supplementary Material

dlad009_Supplementary_Data

## Supplementary data

Tables S1 to S3 are available as Supplementary data at *JAC-AMR* Online.

## References

[1] DeFife R , Scheetz MH, Feinglass JM et al Effect of differences in MIC values on clinical outcomes in patients with bloodstream infections caused by gram-negative organisms treated with levofloxacin. Antimicrob Agents Chemother 2009; 53: 1074–9. 10.1128/AAC.00580-08 19075047 PMC2650520

[2] O’Donnell JN , Rhodes NJ, Biehle LR et al Assessment of mortality stratified by meropenem minimum inhibitory concentration in patients with Enterobacteriaceae bacteraemia: a patient-level analysis of published data. Int J Antimicrob Agents. 2020; 55: 105849. 10.1016/j.ijantimicag.2019.11.006 31770628

[3] Ratliff AR , Gentry CA, Williams RJ 2nd. A propensity score-matched analysis of the impact of minimum inhibitory concentration on mortality in patients with *Pseudomonas aeruginosa* bacteremia treated with cefepime or ceftazidime. Diagn Microbiol Infect Dis 2017; 87: 376–81. 10.1016/j.diagmicrobio.2016.12.016 28087171

[4] Evans R , Pike K, Rogers C et al Modifiable healthcare factors affecting 28-day survival in bloodstream infection: a prospective cohort study. BMC Infect Dis 2020; 20: 545. 10.1186/s12879-020-05262-6 32711452 PMC7382856

[5] CLSI . Methods for Dilution Susceptibility Tests for Bacteria That Grow Aerobically —Eighth Edition: M07. 2009.

[6] EUCAST . Breakpoint tables for interpretation of MICs and zone diameters. Version 9. 2020. https://www.eucast.org/clinical_breakpoints/.

[7] Jann B . Stata tip 8: splitting time-span records with categorical time-varying covariates. Stata J 2004; 4: 221–2. 10.1177/1536867X0400400212

[8] Delgado-Valverde M , Torres E, Valiente-Mendez A et al Impact of the MIC of piperacillin/tazobactam on the outcome for patients with bacteraemia due to Enterobacteriaceae: the Bacteraemia-MIC project. J Antimicrob Chemother 2016; 71: 521–30. 10.1093/jac/dkv362 26538507

[9] Gentry CA , Williams RJ 2nd . A propensity score-matched analysis of the impact of minimum inhibitory concentration on mortality in patients with *Pseudomonas aeruginosa* bacteraemia treated with piperacillin/tazobactam. Int J Antimicrob Agents 2017; 49: 333–8. 10.1016/j.ijantimicag.2016.11.018 28108367

[10] Yoon CH , Bartlett S, Stoesser N et al Mortality risks associated with empirical antibiotic activity in *Escherichia coli* bacteraemia: an analysis of electronic health records. J Antimicrob Chemother 2022; 77: 2536–45. 10.1093/jac/dkac189 35723965 PMC9410673

[11] Jacob JT , DiazGranados CA. High vancomycin minimum inhibitory concentration and clinical outcomes in adults with methicillin-resistant *Staphylococcus aureus* infections: a meta-analysis. Int J Infect Dis 2013; 17: e93–e100. 10.1016/j.ijid.2012.08.005 23089040 PMC3780595

