# Supplementary material for: The relationship between minimum inhibitory concentration and 28 day mortality in patients with a Gram-negative bloodstream infection: an analysis of data from a cohort study (BSI-FOO)
Source: JAC Antimicrob Resist. 2023 Feb 1;5(1):dlad009. doi: 10.1093/jacamr/dlad009 (PMC9891342; doi:10.1093/jacamr/dlad009)
Supplement: dlad009_Supplementary_Data [file dlad009_supplementary_data.pdf]

The relationship between minimum inhibitory concentration and 28-day mortality in patients with a Gram-negative bloodstream infection: an analysis of data from a cohort study (BSI-FOO)

Supplementary material

Supplemental tables

Supplementary Table S1 Variables included in model used to calculate risk score 2
Supplementary Table S2 Baseline characteristics, by MIC/EUCAST ratio category........................... 3
Supplementary Table S3 Antibiotics prescribed, by organism 7

Supplementary Table S1 Variables included in model used to calculate risk score

Type	Factors
Organisational	Admission from nursing or care home
Organism / infection	Source of infection (CDC criteria)
Patient measures	Age
	Weight (kg)
Patient medical history and comorbidities (up to date 0)	Leukaemia within 5 years before date 0
	Solid tumour within 5 years before date 0
	Any other (second) tumour within 5 years before date 0
	Renal support within 7 days before date 0
	Ascites
	Congestive heart failure
	Cerebrovascular disease
	Peptic ulcer disease
	Peripheral vascular disease
	Abscess at time 0
Infection severity measures at or nearest before time 0	Signs
	Mental Disorientation (scale 0-4) at time 0
	Temperature (°C) at time 0
	Systolic blood pressure (mmHg) at time 0
	Blood tests
	eGFR[d] (mL/min/1.73 m^2) at day 0, or nearest within 7 days before
	Serum albumin (g/L) at day0, or nearest within 7 days before
	Bilirubin (total, micromol/L) at day0, or nearest within 7 days before
	Interventions
	Receiving intravenous fluids on day 0, at or before time 0
	Receiving artificial ventilation on day 0, at or before time 0
	Received systemic corticosteroids in 24 hours before time 0

Supplementary Table S2 Baseline characteristics, by MIC/EUCAST ratio category

	MIC/EUCAST breakpoint ratio on last day of follow-up*					
	**<0.125 (S) (n=121)**	**0.125 – <0.25 (S) (n=98)**	**0.25 – <0.5 (S) (n=140)**	**0.5 - <1 (S) (n=90)**	**= 1 (S) (n=36)**	**>1 (R) (n=29)**
**Patient measures**						
Age	72.0 (58.0, 82.0)	71.5 (62.0, 82.0)	75.5 (62.0, 82.0)	75.5 (60.0, 83.0)	75.5 (64.5, 83.5)	66.0 (53.0, 79.0)
Male	63/121 (52.1%)	52/98 (53.1%)	72/140 (51.4%)	37/90 (41.1%)	14/36 (38.9%)	15/29 (51.7%)
Body Mass Index [a]	26.4 (7.7)	24.8 (5.4)	25.6 (7.3)	25.9 (6.9)	26.0 (4.2)	27.1 (5.4)
**Organism**						
E. coli	110/121 (90.9%)	79/98 (80.6%)	72/140 (51.4%)	52/90 (57.8%)	28/36 (77.8%)	22/29 (75.9%)
P. aeruginosa	11/121 (9.1%)	19/98 (19.4%)	68/140 (48.6%)	38/90 (42.2%)	8/36 (22.2%)	7/29 (24.1%)
**Patient medical history**						
Chemotherapy in month before date 0	18/121 (14.9%)	22/98 (22.4%)	34/140 (24.3%)	10/90 (11.1%)	5/36 (13.9%)	4/29 (13.8%)
Any tumour within last 5 years	41/121 (33.9%)	44/98 (44.9%)	67/140 (47.9%)	34/90 (37.8%)	17/36 (47.2%)	11/29 (37.9%)
Surgery requiring overnight stay within 7 days before date 0	7/121 (5.8%)	7/98 (7.1%)	6/140 (4.3%)	5/89 (5.6%)	5/36 (13.9%)	4/29 (13.8%)
Burn requiring admission within 7 days before date 0	0/121 (0.0%)	0/98 (0.0%)	0/140 (0.0%)	0/89 (0.0%)	0/36 (0.0%)	0/29 (0.0%)
Cardiac arrest within 7 days before date 0	1/121 (0.8%)	0/98 (0.0%)	2/140 (1.4%)	1/90 (1.1%)	0/36 (0.0%)	0/29 (0.0%)
Renal support within 7 days before date 0	9/121 (7.4%)	2/98 (2.0%)	8/140 (5.7%)	2/90 (2.2%)	1/36 (2.8%)	5/29 (17.2%)
Myocardial infarction within 7 days before date 0	13/121 (10.7%)	11/98 (11.2%)	12/140 (8.6%)	5/90 (5.6%)	3/36 (8.3%)	6/29 (20.7%)
**Infection severity measures**						
Temperature (°C) at time 0 [b]	38.1 (37.5, 38.7)	38.5 (38.0, 39.1)	38.1 (37.6, 38.6)	38.2 (37.4, 38.8)	38.3 (37.5, 38.6)	38.5 (37.6, 39.0)
INR [c]	1.2 (1.1, 1.3)	1.1 (1.1, 1.5)	1.1 (1.0, 1.3)	1.2 (1.1, 1.3)	1.3 (1.0, 1.5)	1.3 (1.1, 2.3)
eGFR (mL/min/1.73m^2) [d]	49.0 (25.0, 90.0)	62.0 (45.0, 90.0)	60.0 (33.0, 90.0)	53.0 (35.0, 90.0)	41.0 (31.0, 65.0)	51.0 (30.0, 75.0)
Serum Albumin (g/L) [e]	30.9 (8.2)	33.9 (7.2)	32.5 (7.6)	30.8 (7.8)	30.3 (6.3)	29.2 (8.4)
Bilirubin total (umol/L) [f]	12.0 (8.0, 21.5)	14.0 (8.0, 25.5)	12.0 (9.0, 22.0)	16.0 (9.0, 24.0)	11.5 (9.0, 21.5)	12.5 (7.5, 18.5)
Neutrophil count at day 0 or closest (x10^9/L) [g]	10.2 (5.0, 14.0)	8.4 (1.7, 14.1)	9.4 (4.5, 15.6)	11.9 (7.5, 16.6)	10.1 (7.5, 15.6)	11.8 (6.3, 14.4)

	MIC/EUCAST breakpoint ratio on last day of follow-up*					
	**<0.125 (S) (n=121)**	**0.125 – <0.25 (S) (n=98)**	**0.25 – <0.5 (S) (n=140)**	**0.5 - <1 (S) (n=90)**	**= 1 (S) (n=36)**	**>1 (R) (n=29)**
Systolic BP at day 0 or closest (mmHg) [h]	121.9 (23.4)	122.1 (29.9)	117.7 (27.3)	118.4 (28.3)	112.4 (23.0)	117.9 (29.5)
On IV fluids at day 0	53/121 (43.8%)	33/98 (33.7%)	46/140 (32.9%)	31/90 (34.4%)	11/36 (30.6%)	13/29 (44.8%)
On ventilation at day 0	9/121 (7.4%)	6/98 (6.1%)	10/140 (7.1%)	9/89 (10.1%)	1/36 (2.8%)	5/29 (17.2%)
On vasopressor drugs at day 0	9/121 (7.4%)	4/98 (4.1%)	6/140 (4.3%)	5/90 (5.6%)	0/36 (0.0%)	4/29 (13.8%)
Systemic corticosteroids in last 24 hours	14/121 (11.6%)	15/98 (15.3%)	22/140 (15.7%)	9/90 (10.0%)	6/36 (16.7%)	1/29 (3.4%)
EWS score nearest to day 0 [i]	3.0 (1.0, 5.0)	2.0 (1.0, 4.0)	3.0 (1.0, 5.0)	2.0 (1.0, 3.0)	2.0 (1.0, 4.0)	3.0 (1.5, 3.0)
**Patient comorbidities at date 0**						
Congestive heart failure	17/121 (14.0%)	11/98 (11.2%)	18/140 (12.9%)	8/90 (8.9%)	3/36 (8.3%)	1/29 (3.4%)
Peripheral vascular disease	12/121 (9.9%)	9/98 (9.2%)	12/140 (8.6%)	6/90 (6.7%)	4/36 (11.1%)	2/29 (6.9%)
Cerebrovascular disease	24/121 (19.8%)	13/98 (13.3%)	26/140 (18.6%)	16/90 (17.8%)	5/36 (13.9%)	4/29 (13.8%)
Hemiplegia	4/121 (3.3%)	4/98 (4.1%)	7/140 (5.0%)	2/90 (2.2%)	2/36 (5.6%)	1/29 (3.4%)
Dementia	13/121 (10.7%)	12/98 (12.2%)	12/140 (8.6%)	12/90 (13.3%)	2/36 (5.6%)	4/29 (13.8%)
COPD	18/121 (14.9%)	11/98 (11.2%)	19/140 (13.6%)	6/90 (6.7%)	7/36 (19.4%)	4/29 (13.8%)
Connective tissue disease	17/121 (14.0%)	4/98 (4.1%)	11/140 (7.9%)	3/90 (3.3%)	6/36 (16.7%)	2/29 (6.9%)
Peptic ulcer disease	9/121 (7.4%)	8/98 (8.2%)	11/140 (7.9%)	8/90 (8.9%)	2/36 (5.6%)	4/29 (13.8%)
Ascites	5/121 (4.1%)	4/98 (4.1%)	3/140 (2.1%)	0/90 (0.0%)	3/36 (8.3%)	1/29 (3.4%)
Diabetes:						
None	85/121 (70.2%)	81/98 (82.7%)	116/140 (82.9%)	78/90 (86.7%)	28/36 (77.8%)	23/29 (79.3%)
Without organ damage	24/121 (19.8%)	16/98 (16.3%)	19/140 (13.6%)	10/90 (11.1%)	7/36 (19.4%)	6/29 (20.7%)
With organ damage	12/121 (9.9%)	1/98 (1.0%)	5/140 (3.6%)	2/90 (2.2%)	1/36 (2.8%)	0/29 (0.0%)
Child-Pugh score [j]	7.0 (6.0, 8.0)	7.0 (6.0, 7.0)	6.0 (6.0, 7.0)	6.0 (5.0, 7.0)	7.0 (6.0, 8.0)	7.0 (7.0, 8.0)
Charlson score [k]	3.0 (2.0, 5.0)	3.0 (2.0, 4.0)	3.0 (2.0, 4.0)	3.0 (2.0, 4.0)	4.0 (3.0, 5.0)	3.0 (2.0, 4.0)
Abscess at time 0	5/121 (4.1%)	3/98 (3.1%)	1/140 (0.7%)	1/90 (1.1%)	0/36 (0.0%)	2/29 (6.9%)
Infected foreign body at time 0	0/121 (0.0%)	0/98 (0.0%)	1/140 (0.7%)	2/90 (2.2%)	0/36 (0.0%)	0/29 (0.0%)
Surgical prosthesis time 0	1/121 (0.8%)	0/98 (0.0%)	1/140 (0.7%)	0/90 (0.0%)	0/36 (0.0%)	0/29 (0.0%)
**Source of infection**						

	MIC/EUCAST breakpoint ratio on last day of follow-up*					
	**<0.125 (S) (n=121)**	**0.125 – <0.25 (S) (n=98)**	**0.25 – <0.5 (S) (n=140)**	**0.5 - <1 (S) (n=90)**	**= 1 (S) (n=36)**	**>1 (R) (n=29)**
Bone and joint	1/121 (0.8%)	0/98 (0.0%)	2/140 (1.4%)	1/90 (1.1%)	0/36 (0.0%)	0/29 (0.0%)
Eye, ear, nose, throat or mouth	1/121 (0.8%)	0/98 (0.0%)	0/140 (0.0%)	0/90 (0.0%)	0/36 (0.0%)	0/29 (0.0%)
Gastrointestinal system	11/121 (9.1%)	15/98 (15.3%)	16/140 (11.4%)	13/90 (14.4%)	5/36 (13.9%)	8/29 (27.6%)
Line infection - central venous line	0/121 (0.0%)	2/98 (2.0%)	13/140 (9.3%)	1/90 (1.1%)	2/36 (5.6%)	1/29 (3.4%)
Lower respiratory tract	3/121 (2.5%)	4/98 (4.1%)	7/140 (5.0%)	11/90 (12.2%)	3/36 (8.3%)	2/29 (6.9%)
Reproductive tract	1/121 (0.8%)	0/98 (0.0%)	0/140 (0.0%)	2/90 (2.2%)	0/36 (0.0%)	0/29 (0.0%)
Skin and soft tissue	5/121 (4.1%)	3/98 (3.1%)	5/140 (3.6%)	0/90 (0.0%)	0/36 (0.0%)	0/29 (0.0%)
Surgical site infection	2/121 (1.7%)	1/98 (1.0%)	4/140 (2.9%)	4/90 (4.4%)	0/36 (0.0%)	0/29 (0.0%)
Systemic Infection	0/121 (0.0%)	0/98 (0.0%)	1/140 (0.7%)	1/90 (1.1%)	0/36 (0.0%)	1/29 (3.4%)
Urinary tract infection	54/121 (44.6%)	34/98 (34.7%)	54/140 (38.6%)	33/90 (36.7%)	18/36 (50.0%)	11/29 (37.9%)
Site uncertain	43/121 (35.5%)	39/98 (39.8%)	38/140 (27.1%)	24/90 (26.7%)	8/36 (22.2%)	6/29 (20.7%)
**Lines and catheters**						
Central line present at time 0	25/121 (20.7%)	21/98 (21.4%)	33/140 (23.6%)	13/90 (14.4%)	4/36 (11.1%)	8/29 (27.6%)
Peripheral line present at time 0	60/121 (49.6%)	41/98 (41.8%)	65/140 (46.4%)	45/90 (50.0%)	12/36 (33.3%)	14/29 (48.3%)
Urinary catheter present at time 0	48/121 (39.7%)	27/98 (27.6%)	43/140 (30.7%)	38/90 (42.2%)	8/36 (22.2%)	12/29 (41.4%)
**Organisational factors**						
Centre:						
A	15/121 (12.4%)	10/98 (10.2%)	10/140 (7.1%)	3/90 (3.3%)	2/36 (5.6%)	1/29 (3.4%)
B	31/121 (25.6%)	12/98 (12.2%)	22/140 (15.7%)	18/90 (20.0%)	11/36 (30.6%)	8/29 (27.6%)
C	36/121 (29.8%)	54/98 (55.1%)	62/140 (44.3%)	47/90 (52.2%)	14/36 (38.9%)	13/29 (44.8%)
D	26/121 (21.5%)	11/98 (11.2%)	20/140 (14.3%)	12/90 (13.3%)	4/36 (11.1%)	3/29 (10.3%)
E	13/121 (10.7%)	11/98 (11.2%)	26/140 (18.6%)	10/90 (11.1%)	5/36 (13.9%)	4/29 (13.8%)

Notes: *Date and time 0 = date/time of sampling for blood culture*

Data are presented as median (IQR), mean (SD) or n (%)

** Day 7 or date of death if earlier*

Missing data (<0.125, 0.125 – <0.25, 0.25 – <0.5, 0.5 - <1, = 1, >1)

[a] Data missing for 252 patients (63, 46, 61, 44, 21, 17)

[b] *Data missing for 5 patients (4, 0, 0, 0, 0, 1)*
[c] *Data missing for 299 patients (63, 56, 85, 54, 20, 21)*
[d] *Data missing for 43 patients (8, 12, 9, 9, 3, 2)*
[e] *Data missing for 67 patients (14, 21, 14, 11, 4, 3)*
[f] *Data missing for 87 patients (21, 22, 16, 19, 4, 5)*
[g] *Data missing for 39 patients (4, 11, 10, 7, 4, 3)*
[h] *Data missing for 81 patients (16, 14, 21, 19, 5, 6)*
[i] *Data missing for 205 patients (59, 32, 59, 29, 13, 13)*
[j] *Data missing for 321 patients (70, 61, 90, 56, 21, 23)*
[k] *Data missing for 124 patients (30, 29, 25, 25, 6, 9)*
Abbreviations: *BP=Blood pressure, COPD=Chronic obstructive pulmonary disease, eGFR=Estimated glomerular filtration rate, EWS=Early warning score, INR=International normalised ratio, MIC=Minimum inhibitory concentration, IQR=Interquartile range, IV=Intravenous, SMD=Standardised mean difference, SD= Standard deviation*

Supplementary Table S3 Antibiotics prescribed, by organism

Antibiotic	*E. coli* & *Klebsiella* (n=353)	*P. aeruginosa (n=151)*	Overall (n=514)
Amoxicillin	49/353 (13.9%)	0/151 (0.0%)	49/514 (9.5%)
Co-amoxiclav	154/353 (43.6%)	0/151 (0.0%)	154/514 (30.0%)
Piperacillin & Tazobactam	257/353 (72.8%)	122/151 (80.8%)	379/514 (73.7%)
Cefotaxime	1/353 (0.3%)	0/151 (0.0%)	1/514 (0.2%)
Ceftazidime	0/353 (0.0%)	15/151 (9.9%)	15/514 (2.9%)
Ceftriaxone	4/353 (1.1%)	0/151 (0.0%)	4/514 (0.8%)
Ertapenem	7/353 (2.0%)	0/151 (0.0%)	7/514 (1.4%)
Meropenem	108/353 (30.6%)	40/151 (26.5%)	148/514 (28.8%)
Gentamicin	90/353 (25.5%)	61/151 (40.4%)	151/514 (29.4%)
Tobramycin	1/353 (0.3%)	0/151 (0.0%)	1/514 (0.2%)
Colistin	0/353 (0.0%)	5/151 (3.3%)	5/514 (1.0%)
Ciprofloxacin	54/353 (15.3%)	29/151 (19.2%)	83/514 (16.1%)
Levofloxacin	3/353 (0.8%)	1/151 (0.7%)	4/514 (0.8%)

Notes: *Data are presented as n (%)*
